# How much European prescribing physicians know about invasive fungal infections management?

**DOI:** 10.1186/s12879-015-0809-z

**Published:** 2015-02-21

**Authors:** Maricela Valerio, Antonio Vena, Emilio Bouza, Nanna Reiter, Pierluigi Viale, Marcel Hochreiter, Maddalena Giannella, Patricia Muñoz, on behalf the COMIC study group (Collaborative group on Mycosis)

**Affiliations:** Department of Clinical Microbiology and Infectious Diseases, Hospital General Universitario Gregorio Marañón, Madrid, Spain; Instituto de Investigación Sanitaria Gregorio Marañón, Madrid, Spain; Department of Medicine, Facultad de Medicina, Universidad Complutense de Madrid, Madrid, Spain; Intensive Care Unit, Rigshospitalet, Copenhagen University Hospital, Copenhagen, Denmark; Department of Medical and Surgical Sciences, S. Orsola-Malpighi Hospital, Alma Mater Studiorum, University of Bologna, Bologna, Italy; Department of Anesthesiology and Intensive Care Medicine, University Hospital of Heidelberg, Heidelberg, Germany

**Keywords:** Antifungal use, Invasive aspergillosis, Candins, Fluconazole, Antifungal stewardship

## Abstract

**Background:**

The use of systemic antifungal agents has increased in most tertiary care centers. However, antifungal stewardship has deserved very little attention. Our objective was to assess the knowledge of European prescribing physicians as a first step of an international program of antifungal stewardship.

**Methods:**

Staff physicians and residents of 4 European countries were invited to complete a 20-point questionnaire that was based on current guidelines of invasive candidiasis and invasive aspergillosis.

**Results:**

121 physicians (44.6% staff, 55.4% residents) from Spain 53.7%, Italy 17.4%, Denmark 16.5% and Germany 12.4% completed the survey. Hospital departments involved were: medical 51.2%, ICUs 43%, surgical 3.3% and pharmaceutical 2.5%. The mean score of adequate responses (± SD) was 5.8 ± 1.7 points, with statistically significant differences between study site and type of physicians. Regarding candidiasis, 69% of the physicians clearly distinguished colonization from infection and the local rate of fluconazole resistance was known by 24%. The accepted indications of antifungal prophylaxis were known by 38%. Regarding aspergillosis, 52% of responders could differentiate colonization from infection and 42% knew the diagnostic value of galactomannan. Radiological features of invasive aspergillosis were well recognized by 58% of physicians and 57% of them were aware of the antifungal considered as first line treatment. However, only 37% knew the recommended length of therapy.

**Conclusions:**

This simple, easily completed questionnaire enabled us to identify some weakness in the knowledge of invasive fungal infection management among European physicians. This survey could serve as a guide to design a future tailored European training program.

**Electronic supplementary material:**

The online version of this article (doi:10.1186/s12879-015-0809-z) contains supplementary material, which is available to authorized users.

## Background

Invasive fungal infections (IFIs), mainly invasive candidiasis (IC) and pulmonary aspergillosis (IPA), are a major clinical problem due to its high morbidity and mortality and affect many different patient populations cared by a large number of physicians in tertiary care hospitals. The difficulties for establishing a proven diagnosis, the better tolerance of new antifungal drugs and the demonstrated impact of early therapy have led to an extended use of empirical antifungal therapies (AF), mainly in critical and surgical patients. A recent cross sectional cohort study showed that systemic antifungal therapy was administered to 7% of all patients admitted to an intensive care unit (ICU), with only one-third of them having a documented IFI [1].

Previous studies have shown that inappropriate use of antifungal drugs may reach 67-74% in tertiary care hospitals [2-5]. The implementation of antifungal stewardship policies based on first instances on continuous education of healthcare workers may be a partial solution to this problem [6]. However, there are no multicenter studies evaluating the gaps in knowledge on diagnosis and treatment of IFIs and on compliance with current guidelines in European prescribing physicians.

The aim of this study was to assess knowledge of potential AF prescribers in order to design and apply operative training strategies for European physicians, which will subsequently affect behavioral changes and lead to a more appropriate use of antifungal agents.

## Methods

### Study design and setting

We performed a cross-sectional multicenter survey evaluating European prescribing physicians’ attitudes and knowledge about diagnosis and treatment of invasive candidiasis and aspergillosis, and the compliance with current IFI international guidelines [7-9]. Five European tertiary care hospitals participated in the study: Hospital General Universitario Gregorio Marañón, Madrid, Spain; University Hospital of Heidelberg, Heidelberg, Germany; Rigshospitalet, Copenhagen University Hospital, Denmark; Hospital “Sant’ Orsola-Malpighi” at Bologna, Italy and Hospital “Lazzaro Spallanzani” at Rome, Italy. Hospital General Universitario Gregorio Marañón served as the coordinating centre.

Attending physicians and residents, working in areas typically associated with the largest consumption of antifungal drugs (i.e. Hematology, Oncology, Internal Medicine, Surgical wards and, ICUs), were invited to participate to the survey. During the study period (February-March 2013), a responsible investigator for each centre (M.V.; M.H.; N.R.; P.V.; M.G.) personally invited all physicians belonging to the departments with larger antifungal consumption to answer the questionnaire. The participation to the survey was voluntary and if the physician agreed, they were given 20 minutes to complete the test. The questionnaire was personally collected and subsequently sent through email/fax to the coordinating centre by responsible investigators. No incentives were used to perform the survey and consultation of any medical support (i.e. books, apps and websites) was forbidden.

### Questionnaire

According to current international guidelines [7-9], a questionnaire was developed by the steering committee of the Collaboration in Mycology Study Group (COMIC), a multidisciplinary team including infectious disease and clinical microbiology physicians, pharmacologist and different medical and surgical specialities. The questionnaire was anonymously completed and included 20 multiple-choice questions assessing diagnosis and management of IFI (see Additional file 1). Questions targeted to evaluate the inadequate indication of antifungals, such as the clinical interpretation of positive cultures or current recommendations for pre-emptive therapy, were specifically included. Each correct answer was scored as 0.5 points and each incorrect answer as 0 points. Accordingly, the maximum score was 10 points. We also collect information regarding age, sex, department to which physicians belonged and post- graduate years.

In order to evaluate the readability, the comprehensibility, and the length, the survey was first tested among a total of 20 physicians working at the Hospital General Universitario Gregorio Marañón, Madrid. The study was approved by the Ethics Committee of the coordinating center (Comité ético de Investigación Clínica del Hospital General Universitario Gregorio Marañón). Local centers did not consider necessary further approval. Participating physicians’s consent was obtained by local coordinators in order to use the survey results for research purposes. This study was partially supported by the PROMULGA II Project, Instituto de Salud Carlos III (grant number PI13/01148).

### Statistical analysis

Our endpoint was the median knowledge score obtained by the physicians prescribing antifungals in tertiary care institutions. We also decided to compare the performance of different groups of physicians (residents/fellows vs. staff physicians, hospital department: ICUs, medical, surgical and pharmaceutical departments) in the different countries. Haematology and oncology physicians were included in the medical group. Not all physicians answered each questions and, consequently numbers were adjusted as appropriate for each question. The qualitative variables appear with their frequency distribution. The quantitative variables are summarized as the mean and SD when they had a normal distribution and median with range (minimum-maximum) when they had a non-normal distribution. In order to compare how scores differ according to participants’ characteristics we used the parametric t test or the non-parametric ANOVA test. Multiple lineal regression analysis was performed to detect differences in knowledge between different departments, physician categories, and country after adjusting by sex and postgraduate education. Statistical significant was set at p ≤ 0.05. Statistical analysis was made using SPSS® 18.0(SPSS, Chicago, IL, USA).

## Results

One hundred and twenty-one physicians fulfilled the questionnaire. Demographic characteristics of participants and mean score obtained according to sex, type of departments and country are summarized in Table 1. Mean age and years of clinical practice after post-graduate education of participating physicians were 36.5 and 10.6 respectively. Overall, 54.5% of the physicians were women and the mean score of adequate response was 5.8 ± 1.7 points. When scores were compared according to different variables (Table 1) a higher significant mean score was associated with staff physicians (p = 0.01), medical departments (*p* = 0.01) and with Italians respondents (p < 0.01).

**Table 1:** **Demographics characteristics of participants and mean score obtained according to sex, type of physicians, medical departments and country**

**Variable**	**N (%)**	**Score (Mean ± SD)**	***P*** **value**
**Demographics characteristics**			
**Age**			
<30	40 (33.1)	5.3 ± 1.4	
≥30	81 (66.9)	6.1 ± 1.8	0.01
<40	75 (62)	5.3 ± 1.4	0.12
≥40	46 (38)	6.1 ± 1.8	
**Years of practice (mean ± SD)**			
<1	19 (15.8)	5 ± 0.9	
≥1	102 (84.2)	6 ± 1.7	0.01
<**5**	33 (27.8)	5 ± 1.3	<0.001
**≥5**	88 (72.7)	6.2 ± 1.7	
**Sex**			
Female	66 (54.5)	5.9 ± 1.6	0.52
Male	55 (45.5)	5.7 ± 1.8	
**Physicians’ category**			
Residents	67 (55.4)	5.5 ± 1.6	0.01
Staff physicians	54 (44.6)	6.3 ± 1.8	
**Type of departments** ^**(a)**^			
Medical	62 (51.2)	6.2 ± 1.9	
Intensive care units	52 (43)	5.5 ± 1.4	0.01
Surgical	4 (3.3)	5.5 ± 1.5	
Pharmaceutical	3 (2.5)	5.5 ± 0.5	
**Country** ^**(b)**^			
Spain	65 (53.7)	5.6 ± 1.7	
Italy	21 (17.4)	7.3 ± 1.4	<0.01
Denmark	20 (16.5)	5.0 ± 1.6	
Germany	15 (12.4)	5.8 ± 1.1	

^(a)^Although statistical differences in mean scores were not found between the different departments in the simple linear regression analysis, they were found between medical departments and the remaining 3 after adjusting for sex, postgraduate education, and physician category. ^(b)^Statistical differences in mean scores were found between Italians responders and the other physicians in the simple linear regression analysis. This difference remains after adjusting for sex, post-graduate education and physician category.

The response rate to the different questions of the survey ranged from 85% to 100. The percentage of adequate answers regarding departments and physician category for each question is showed in Table 2. In order to assess differences between groups only medical and critical departments were considered due to the low proportion of surgeons and pharmaceutical physicians participating to the survey. Overall, 13 out of 20 questions assessed knowledge of candidiasis whereas 7 focused on diagnosis and management of invasive aspergillosis.

**Table 2:** **Percentage of adequate answers regarding department and physician category**

**Question**	**Adequate answer**	**Overall**	**Medical**	**ICU**	**P**	**Residents**	**Staff**	**P**
		**N = 121**	**n = 62**	**N = 52**		**n = 67**	**n = 54**	
Q1.When *Candida* is isolated in a urine culture, choose the answer that best describes what you would do:	Start antifungal treatment only in some cases.	69.4	79	63.5	0.09	62.7	77.8	0.08
Q2.On a patient with mechanical ventilation and a probable VAP a tracheal aspirate culture shows *Candida* sp. Which of the following statements best show your interpretation:	Requires antifungal treatment only if the patient has a high *Candida* score.	42.1	46.5	38.5	0.44	38.8	46.3	0.46
Q3. In which of the following clinical scenarios you would start *Candida* prophylaxis?	AML (Acute Myeloid Leukemia) patients on induction chemotherapy.	38	45.2	32.7	0.18	29.9	48.1	0.05
Q4.In your opinion, the best choice for *Candida* prophylaxis is:.	Fluconazole in most of the cases.	88.4	83.9	92.3	0.25	85.1	92.6	0.25
Q5.In a patient with sepsis possibly caused by a femoral catheter infection, you would prescribe…	Treatment against Gram positive and Gram negative bacteria and yeasts.	41.3	51.6	28.8	0.02	56.7	22.2	<0.001
Q6 .A microbiologist informs you that there are yeasts at the gram stain of a blood culture, so you…	Start antifungal treatment immediately.	81.8	77.4	86.5	0.23	77.6	87	0.23
Q7.In a patient with candidemia, which antifungal would be your first choice before knowing the species of *Candida*?	Candin or Fluconazole.	90.1	88.7	92.3	0.75	88.1	92.6	0.54
Q8 .Choose the right answer among the following statements:	All of the above are true: *Candida glabrata* can be resistant to fluconazole. *Candida krusei* is always resistant to fluconazole. *Candida parapsilosis* is associated to catheter infection. *Candida albicans* is usually susceptible to fluconazole.	75.2	75.8	73.1	0.83	73.1	77.8	0.67
Q9.During the follow-up of candidemic patients, it is advised to:	All of the above are true: Draw blood cultures after 3–7 days of antifungal treatment; exclude infective endocarditis by transesophageal echocardiography; perform an eye fundus examination; consider sequential treatment switching to an oral azole when clinically safe.	83.5	85.5	80.8	0.61	80.6	87	0.46
Q10. In the treatment of candidemia by a fluconazole-susceptible *Candida*, you would usually prescribe:	Fluconazole 400 to 800 mg per day depending on the *Candida* species.	64.5	74.2	53.8	0.03	62.7	66.7	0.7
Q11.Which do you think is the percentage of fluconazole resistance in *Candida* strains isolated from blood cultures at your hospital?	Less than 5%.	24	19.4	28.8	0.27	23.9	24.1	1
Q12.In which of the following scenarios would you choose L-AmB as your first choice?	In unspecified invasive filamentous fungal infection.	47.1	51.6	44.2	0.45	35.8	61.1	<0.006
Q13. Regarding the treatment with azoles and candins, which of the following statements is true:	Candins can be used as empirical treatment before knowing the yeast antifungal susceptibility.	67.8	64.5	75	0.30	53.7	85.2	<0.001
Q14.When isolating *Aspergillus* spp. in a respiratory sample, you would consider:	Treatment in patients who fulfilled criteria of proven or probable invasive aspergillosis	52.1	74.2	26.9	<0.01	49.3	55.6	0.58
Q15.Which of the following statements regarding the Galactomannan test is false:	It can only be performed in serum samples.	42	50.8	35.3	0.13	36.9	48.1	0.26
Q16.Which of the following are considered invasive aspergillosis radiological findings?	All of the above are true: Presence of dense, well-circumscribed lesions with or without a halo sign in a thoracic CT scanner; presence of a cavity in a thoracic CT scanner; presence of an air-crescent sign in a thoracic CT scanner; sinusitis.	58.7	67.7	51.9	0.12	59.7	57.4	0.85
Q17.In a patient with invasive pulmonary aspergillosis, which antifungal treatment would you choose before having the antifungal susceptibility data?	Voriconazole	57	56.5	61.5	0.70	43.3	74.1	<0.001
Q18.In your opinion, which are the indications of combined antifungal therapy in invasive aspergillosis?	It is recommended as rescue therapy when previous antifungal treatment has failed.	38.8	43.5	30.8	0.17	32.8	46.3	0.14
Q19.What is your opinion concerning the measurement of antifungal levels?	All of the above are true: Up-to-date guidelines do not recommend its systematical determination; it can be useful to identify azoles under-dosed patients; there is no indication to determine serum levels of L-AmB; it can help to identify azoles related toxicity.	62	67.7	55.8	0.24	56.7	68.5	0.19
Q20.In your opinion, which would be the proper length of treatment of aspergillosis in a solid organ recipient	A minimum of 6 to 12 weeks.	36.7	37.1	33.3	0.7	34.8	38.9	0.7

**VAP** (Ventilator Associated Pneumonia).

### Candidiasis

A low proportion of physicians clearly distinguished infection from **colonization** both in the urinary tract (69.4%) (Q1) and in the respiratory tract (42.1%) (Q2). The accepted indications of antifungal **prophylaxis** (Q3) were known by 38% of the participants. However, the majority of the physicians (88.4%) knew that fluconazole is, in most of the cases, the recommended antifungal agent for prophylaxis (Q4).

Regarding **empirical therapy**, only 41.3% of the physicians were aware of the recommendation to initiate antifungal therapy in a clinical scenario of sepsis with a suspected portal of entry in a femoral catheter (Q5). Comparing the rate of adequate answers for Q5, statistical differences could be detected both among different departments, as for physicians’ category. In fact, for the management of such infection approximately one third of the intensivists and staff clinicians would prescribe only empirical treatment against gram positive and gram negative bacteria, completely omitting anti-fungal coverage.

As for **targeted therapy** (Q6), 81.8% started treatment immediately after knowing that yeast was recovered from blood cultures, and 90.1% of the physicians choose the correct/recommended antifungal treatment (Q7).

The majority of the prescribing physicians (75.2%) could identify the specific characteristics of **non-*albicans Candida*species**, including its potential for azole resistance (Q8) and 83.5% recognized the need to consider infectious endocarditis and endophtalmitis in a patient with candidemia, and the importance of obtaining blood cultures during the follow up in order to discard persistent candidemia or treatment failure (Q9).

Sixty-four percent of physicians knew the correct **dosage** of fluconazole (800 mg as loading dose followed by 400 mg/day) (Q10), and the local rate of fluconazole resistance was known only by 24% (it was generally overestimated) (Q11). Finally, regarding the different indications of liposomal amphotericin B (L-AmB), azoles or candins, our questionnaire revealed that only 47.1% of the physicians knew in which scenario L-AmB is the first choice therapy for unspecified IFIs (Q12). A significant proportion of clinicians considered L-AmB as the treatment of choice for aspergillosis (26.5%) and for infections caused by fluconazole-resistant *Candida* species (12%).

Sixty-eight percent answered correctly that candins could be used as empirical treatment of candidemia before knowing the antifungal susceptibility (Q13), but about 10% of respondents preferred voriconazole rather than candins when treating infections due to fluconazole-resistant *Candida*.

### Invasive aspergillosis

Overall 52% of the physicians correctly differentiated respiratory colonization from infection, but only 27% of intensivists would start antifungal treatment if *Aspergillus* spp. was recovered from a respiratory sample, taking into account if the patients fulfilled criteria of proven or probable invasive aspergillosis (p < 0.001) (Q14).

When evaluating **diagnostic criteria**, 42% were acquainted with the use of galactomannan assay as a diagnostic and follow up test (Q15), and more than half of the physicians (58.5%) recognized the radiological features of IA (Q16).

Concerning **therapy** (Q17), 57% of the physicians were aware that voriconazole was the **first line IA** treatment. Many physicians believed that combined antifungal therapy with L-AmB and voriconazole (13.3%) or with voriconazole and caspofungin (11.5%) was accepted as first line therapy for treating IA. L-AmB was believed to be the drug of choice by 14.1% of the physicians (3.5% 10 mg/kg/day and 10.6% 3 mg/kg/day). When asked specifically on the indications of combined treatment for IA, 29% considered it appropriate as initial therapy of neutropenic patients or transplant recipients, and 38.8% only for rescue therapy (Q18).

More than half of physicians (62%) were aware of the clinical benefits of measuring voriconazole and posaconazole **plasma levels** during patient treatment (Q19) and finally, the recommended length of therapy for IA according to current guidelines was only known by 36.7% of the physicians – 29% wrongly considered that 4 to 6 weeks were enough to treat most IA episodes (Q20).

## Discussion

We report the first multicenter European study assessing physicians’ knowledge about current recommendations on diagnosis and treatment of IFI. We could demonstrate that even frequent prescribers have a significant need of continuous education. Most common mistakes lead to antifungal over consumption, since many physicians erroneously treat fungal colonization, use unnecessary high doses of L-AmB and administered combined antifungal therapy with no supporting scientific evidence.

Our survey corroborates gaps in both diagnosis and management of IFI in 5 institutions of 4 European countries. Physicians have problems for **differentiating colonization from infection** when *Candida* spp. is isolated in urine or in a tracheal aspirate, which could lead to an over prescription of antifungals. In a prospective study conducted in a Thai tertiary care setting, Sutepvarnon et al. demonstrated a positive correlation between isolation of *Candida* species from urine and unnecessary treatment [5]. Other common scenario of avoidable over treatment is the isolation of *Candida* in respiratory secretions which should not be the only indication for starting antifungal therapy [7,10].

Another aspect deserving attention is the lack of knowledge of the current indications of **antifungal prophylaxis and empirical treatment** for invasive candidiasis. Almost 50% of physicians would initiate *Candida* prophylaxis in every ICU patient colonized by *Candida* spp. or in the clinical scenario of an ICU patient having an urinary indwelling catheter, central venous catheter and recent surgery, without taking into consideration other risk factors or clinical manifestations. A probable reason for a such inadequate management could be related to the results of at least four prospective studies of ICU and surgical patients suggesting a reduction in *Candida* infection and mortality under fluconazole prophylaxis [11-14]. However, since the universal administration of antifungal prophylaxis remains an inefficient strategy that may increase subsequent azole-resistance or non-*albicans* candidemia [15-17], it is currently warranted only in selected ICU patients at highest risk (>10%) of invasive candidiasis [7,18].

On the other hand, we observed a partial impact on medical practice of the articles demonstrating the importance of an **early initiation of targeted antifungal treatment** when there is a clinical suspicion of invasive candidiasis [19-21]. Unfortunately, about 20% of physicians delay the start of antifungal treatment after being informed of positive blood cultures, whereas the critical window of opportunity for antifungal initiation appears to be 12–24 hours following the first positive blood cultures were drawn [20,22]. Moreover, we reported that 60% of physicians (staff more frequently than residents and intensivists more frequently than medical physicians) failed to identify the indications of antifungal treatment in patients with suspicion of **catheter related infection** [23].

However, the clinical outcome in IFI related sepsis is not only related to adequacy and timeliness of antifungal administration, but also on appropriate dosing, all these factors associated with length of hospital stay, health care costs, morbidity and mortality [20,22,24]. We found that another critical point regarding knowledge of *Candida* infections management is the **appropriate fluconazole dosage,** that was correctly known by only 62% of the physicians. In a retrospective cohort study performed by Labelle et al., inadequate initial fluconazole dose was prescribed in about half of the critical patients with invasive candidiasis and it was associated with an increased risk of in-hospital mortality [25]. Indeed, failure to achieve pharmacodynamic targets for fluconazole has been associated with worse outcomes [26-29]. For this reason, to obtain clinical success, dose of fluconazole should be tailored to achieve AUC/MIC ratios of at least 25 (using Clinical and Laboratory Standards Institute MIC methodology) [30] or 100 (using European Committee on Antimicrobial Susceptibility Testing MIC methodology). These targets usually require 6 mg/kg/day (following 12 mg/kg loading) for susceptible isolates or 12 mg/kg/day for *C. glabrata* or other isolates with MICs of 16-32 mg/L [7].

Most physicians were not aware of their local incidence of azoles resistance in *Candida*, which could enhance the prescription of broad-spectrum antifungals. Although non-*albicans* strains have clearly increased, in many European centers the rate of fluconazole resistance is still less than 5% [31-34].

More surprisingly to us was to find that 12% and 9.2% of physicians would select L-AmB and voriconazole, respectively, instead of a candin to treat these fluconazole-resistant *Candida* infections. L-AmB should be restricted to selected cases of intra-abdominal candidiasis [35,36] or intolerance to other antifungal agents due to its potential toxicity and higher cost, and voriconazole should be limited to step-down oral therapy for selected cases of candidiasis due to *C. krusei* or voriconazole-susceptible *C. glabrata* [7]. Finally, we would like to stress that there are differences among published guidelines regarding some diagnostic and therapeutic aspects of candidiasis that need to be clarified for the sake of a more clear understanding by the prescribing physicians [37].

Regarding IA, problems to **differentiate colonization from infection** were also evident. As previously demonstrated only 22.3% of *Aspergillus* isolates from respiratory tract corresponds to probable/proven IA episodes. For this reason, Bouza et al. proposed a prediction score taking into account the procedure used to obtain the sample, the presence of leukaemia, neutropenia or the use of corticoids to help clinicians in the interpretation of *Aspergillus* cultures [38].

Another problem was that a high percentage of the prescribing physicians still consider **L-AmB as the first line therapy for IA**, whereas the largest prospective, randomized trial for the treatment of IA demonstrated that voriconazole was superior to D-AmB [8,39]. Furthermore, 3.5% of the physicians continue to believe that **high doses of L-AmB** are necessary to treat IA, ignoring the results of the AmBiLoad Trial [40,41]. The efficacy of voriconazole was further demonstrated in paediatric and adult patients receiving voriconazole for treatment of IA who were refractory or intolerant to conventional antifungal therapy [8,39,42,43]. The role of combination therapy as primary or salvage therapy is uncertain and it is not actually recommended in international guidelines [8].

The majority of physicians ignored that current guidelines recommend that treatment of IA should be continued for a minimum of 6–12 weeks and that voriconazole plasma levels should be monitored during treatment to avoid toxicity and therapeutic failure [44-47].

Finally, we found differences in knowledge between experimented physicians and residents. As we expected, the haematologists and infectious disease specialists (medical departments), are more proficient in the use of antifungal therapy. However, ICU physicians that are directly responsible for many pre-emptive and empirical antifungal prescriptions did not score so well comparatively. Interestingly, Apisarnthanarak et al. reported that 44% and 31% of inappropriate antifungal use was detected in medical departments and ICUs respectively, and 15% in surgical departments [4].

The primary limitations of our study include the selection of physicians that more frequently prescribe antifungal agents and the hospitals for the questionnaire. This means that the results could be worse if all the physicians of all institutions had been offered to participate. We also included a heterogeneous group of physicians who are mainly involved in different fields of IFI infection and only addressed knowledge in *Candida* and *Aspergillus* invasive infections. Finally, although the response rate to different questions was high (85-100%), potential bias could result due to non-response to specific questions.

Given the findings of our study assessing the basal knowledge of antifungal prescribers in 5 European institutions, operative attempts to ameliorate the inappropriate use of medications should be performed. We believe that these European interventions should be based on: 1) Educational programs focusing on correct drugs and dose administration of antifungals; 2) Improvement in appropriate clinical interpretation of fungal isolates; 3) Establishment of antifungal management programs that incorporate infectious diseases specialists that as previously documented [5,48] are important in order to improve quality of care while optimizing hospital costs and antifungal use.

## Conclusions

In conclusion, a simple survey enabled us to assess the knowledge and practice of European prescribing physicians in important aspects of diagnosis, prophylaxis and antifungal treatment of IFIs. This study has revealed that there are serious lacks in knowledge in this area that requires a tailored educational program as a first step of an international antifungal stewardship implementation.

## Additional file

Additional file 1:
**Consists in the 20- point survey administered to participants.**

## References

[1] Azoulay E, Dupont H, Tabah A, Lortholary O, Stahl JP, Francais A (2012). Systemic antifungal therapy in critically ill patients without invasive fungal infection*. Crit Care Med.

[2] Valerio M, Rodriguez-Gonzalez CG, Munoz P, Caliz B, Sanjurjo M, Bouza E (2014). Evaluation of antifungal use in a tertiary care institution: antifungal stewardship urgently needed. J Antimicrob Chemother.

[3] Nivoix Y, Launoy A, Lutun P, Moulin JC, Phai Pang KA, Fornecker LM (2012). Adherence to recommendations for the use of antifungal agents in a tertiary care hospital. J Antimicrob Chemother.

[4] Apisarnthanarak A, Yatrasert A, Mundy LM (2010). Impact of education and an antifungal stewardship program for candidiasis at a Thai tertiary care center. Infect Control Hosp Epidemiol.

[5] Sutepvarnon A, Apisarnthanarak A, Camins B, Mondy K, Fraser VJ (2008). Inappropriate use of antifungal medications in a tertiary care center in Thailand: a prospective study. Infect Control Hosp Epidemiol.

[6] Dellit TH, Owens RC, McGowan JE, Gerding DN, Weinstein RA, Burke JP (2007). Infectious Diseases Society of America and the Society for Healthcare Epidemiology of America guidelines for developing an institutional program to enhance antimicrobial stewardship. Clin Infect Dis.

[7] Pappas PG, Kauffman CA, Andes D, Benjamin DK, Calandra TF, Edwards JE (2009). Clinical practice guidelines for the management of candidiasis: 2009 update by the Infectious Diseases Society of America. Clin Infect Dis.

[8] Walsh TJ, Anaissie EJ, Denning DW, Herbrecht R, Kontoyiannis DP, Marr KA (2008). Treatment of aspergillosis: clinical practice guidelines of the Infectious Diseases Society of America. Clin Infect Dis.

[9] Cornely OA, Bassetti M, Calandra T, Garbino J, Kullberg BJ, Lortholary O (2012). ESCMID* guideline for the diagnosis and management of Candida diseases 2012: non-neutropenic adult patients. Clin Microbiol Infect.

[10] El-Ebiary M, Torres A, Fabregas N, de la Bellacasa JP, Gonzalez J, Ramirez J (1997). Significance of the isolation of Candida species from respiratory samples in critically ill, non-neutropenic patients. An immediate postmortem histologic study. Am J Respir Crit Care Med.

[11] Eggimann P, Francioli P, Bille J, Schneider R, Wu MM, Chapuis G (1999). Fluconazole prophylaxis prevents intra-abdominal candidiasis in high-risk surgical patients. Crit Care Med.

[12] Garbino J, Lew DP, Romand JA, Hugonnet S, Auckenthaler R, Pittet D (2002). Prevention of severe Candida infections in nonneutropenic, high-risk, critically ill patients: a randomized, double-blind, placebo-controlled trial in patients treated by selective digestive decontamination. Intensive Care Med.

[13] Jacobs S, Price Evans DA, Tariq M, Al Omar NF (2003). Fluconazole improves survival in septic shock: a randomized double-blind prospective study. Crit Care Med.

[14] Pelz RK, Hendrix CW, Swoboda SM, Diener-West M, Merz WG, Hammond J (2001). Double-blind placebo-controlled trial of fluconazole to prevent candidal infections in critically ill surgical patients. Ann Surg.

[15] Playford EG, Eggimann P, Calandra T (2008). Antifungals in the ICU. Curr Opin Infect Dis.

[16] Slavin MA, Sorrell TC, Marriott D, Thursky KA, Nguyen Q, Ellis DH (2010). Candidaemia in adult cancer patients: risks for fluconazole-resistant isolates and death. J Antimicrob Chemother.

[17] Garnacho-Montero J, Diaz-Martin A, Garcia-Cabrera E, Ruiz Perez de Pipaon M, Hernandez-Caballero C, Aznar-Martin J (2010). Risk factors for fluconazole-resistant candidemia. Antimicrob Agents Chemother.

[18] Ostrosky-Zeichner L, Sable C, Sobel J, Alexander BD, Donowitz G, Kan V (2007). Multicenter retrospective development and validation of a clinical prediction rule for nosocomial invasive candidiasis in the intensive care setting. Eur J Clin Microbiol Infect Dis.

[19] Kumar A, Ellis P, Arabi Y, Roberts D, Light B, Parrillo JE (2009). Initiation of inappropriate antimicrobial therapy results in a fivefold reduction of survival in human septic shock. Chest.

[20] Morrell M, Fraser VJ, Kollef MH (2005). Delaying the empiric treatment of candida bloodstream infection until positive blood culture results are obtained: a potential risk factor for hospital mortality. Antimicrob Agents Chemother.

[21] Kollef M, Micek S, Hampton N, Doherty JA, Kumar A (2012). Septic shock attributed to Candida infection: importance of empiric therapy and source control. Clin Infect Dis.

[22] Garey KW, Rege M, Pai MP, Mingo DE, Suda KJ, Turpin RS (2006). Time to initiation of fluconazole therapy impacts mortality in patients with candidemia: a multi-institutional study. Clin Infect Dis.

[23] Mermel LA, Allon M, Bouza E, Craven DE, Flynn P, O'Grady NP (2009). Clinical practice guidelines for the diagnosis and management of intravascular catheter-related infection: 2009 Update by the Infectious Diseases Society of America. Clin Infect Dis.

[24] Zilberberg MD, Kollef MH, Arnold H, Labelle A, Micek ST, Kothari S (2010). Inappropriate empiric antifungal therapy for candidemia in the ICU and hospital resource utilization: a retrospective cohort study. BMC Infect Dis.

[25] Labelle AJ, Micek ST, Roubinian N, Kollef MH (2008). Treatment-related risk factors for hospital mortality in Candida bloodstream infections. Crit Care Med.

[26] Clancy CJ, Yu VL, Morris AJ, Snydman DR, Nguyen MH (2005). Fluconazole MIC and the fluconazole dose/MIC ratio correlate with therapeutic response among patients with candidemia. Antimicrob Agents Chemother.

[27] Pai MP, Turpin RS, Garey KW (2007). Association of fluconazole area under the concentration-time curve/MIC and dose/MIC ratios with mortality in nonneutropenic patients with candidemia. Antimicrob Agents Chemother.

[28] Baddley JW, Patel M, Bhavnani SM, Moser SA, Andes DR (2008). Association of fluconazole pharmacodynamics with mortality in patients with candidemia. Antimicrob Agents Chemother.

[29] Rodriguez-Tudela JL, Almirante B, Rodriguez-Pardo D, Laguna F, Donnelly JP, Mouton JW (2007). Correlation of the MIC and dose/MIC ratio of fluconazole to the therapeutic response of patients with mucosal candidiasis and candidemia. Antimicrob Agents Chemother.

[30] Pfaller MA, Diekema DJ, Sheehan DJ (2006). Interpretive breakpoints for fluconazole and Candida revisited: a blueprint for the future of antifungal susceptibility testing. Clin Microbiol Rev.

[31] Tortorano AM, Kibbler C, Peman J, Bernhardt H, Klingspor L, Grillot R (2006). Candidaemia in Europe: epidemiology and resistance. Int J Antimicrob Agents.

[32] Guinea J, Pelaez T, Rodriguez-Creixems M, Torres-Narbona M, Munoz P, Alcala L (2009). Empirical treatment of candidemia in intensive care units: fluconazole or broad-spectrum antifungal agents?. Med Mycol.

[33] Munoz P, Fernandez-Turegano CP, Alcala L, Rodriguez-Creixems M, Pelaez T, Bouza E (2002). Frequency and clinical significance of bloodstream infections caused by C albicans strains with reduced susceptibility to fluconazole. Diagn Microbiol Infect Dis.

[34] Pfaller MA, Messer SA, Woosley LN, Jones RN, Castanheira M (2013). Echinocandin and triazole antifungal susceptibility profiles for clinical opportunistic yeast and mold isolates collected from 2010 to 2011: application of new CLSI clinical breakpoints and epidemiological cutoff values for characterization of geographic and temporal trends of antifungal resistance. J Clin Microbiol.

[35] Bassetti M, Marchetti M, Chakrabarti A, Colizza S, Garnacho-Montero J, Kett DH (2013). A research agenda on the management of intra-abdominal candidiasis: results from a consensus of multinational experts. Intensive Care Med.

[36] Pea F (2013). Current pharmacological concepts for wise use of echinocandins in the treatment of Candida infections in septic critically ill patients. Expert Rev Anti Infect Ther.

[37] Deshpande A, Gaur S, Bal AM (2013). Candidaemia in the non-neutropenic patient: a critique of the guidelines. Int J Antimicrob Agents.

[38] Bouza E, Guinea J, Pelaez T, Perez-Molina J, Alcala L, Munoz P (2005). Workload due to Aspergillus fumigatus and significance of the organism in the microbiology laboratory of a general hospital. J Clin Microbiol.

[39] Herbrecht R, Denning DW, Patterson TF, Bennett JE, Greene RE, Oestmann JW (2002). Voriconazole versus amphotericin B for primary therapy of invasive aspergillosis. N Engl J Med.

[40] Cornely OA, Maertens J, Bresnik M, Ebrahimi R, Ullmann AJ, Bouza E (2007). Liposomal amphotericin B as initial therapy for invasive mold infection: a randomized trial comparing a high-loading dose regimen with standard dosing (AmBiLoad trial). Clin Infect Dis.

[41] Munoz P, Guinea J, Narbona MT, Bouza E (2008). Treatment of invasive fungal infections in immunocompromised and transplant patients: AmBiLoad trial and other new data. Int J Antimicrob Agents.

[42] Walsh TJ, Pappas P, Winston DJ, Lazarus HM, Petersen F, Raffalli J (2002). Voriconazole compared with liposomal amphotericin B for empirical antifungal therapy in patients with neutropenia and persistent fever. N Engl J Med.

[43] Perfect JR, Marr KA, Walsh TJ, Greenberg RN, DuPont B, de la Torre-Cisneros J (2003). Voriconazole treatment for less-common, emerging, or refractory fungal infections. Clin Infect Dis.

[44] Smith J, Andes D (2008). Therapeutic drug monitoring of antifungals: pharmacokinetic and pharmacodynamic considerations. Ther Drug Monit.

[45] Pasqualotto AC, Xavier MO, Andreolla HF, Linden R (2010). Voriconazole therapeutic drug monitoring: focus on safety. Expert Opin Drug Saf.

[46] Pascual A, Csajka C, Buclin T, Bolay S, Bille J, Calandra T (2012). Challenging recommended oral and intravenous voriconazole doses for improved efficacy and safety: population pharmacokinetics-based analysis of adult patients with invasive fungal infections. Clin Infect Dis.

[47] Pascual A, Calandra T, Bolay S, Buclin T, Bille J, Marchetti O (2008). Voriconazole therapeutic drug monitoring in patients with invasive mycoses improves efficacy and safety outcomes. Clin Infect Dis.

[48] McQuillen DP, Petrak RM, Wasserman RB, Nahass RG, Scull JA, Martinelli LP (2008). The value of infectious diseases specialists: non-patient care activities. Clin Infect Dis.

