# Supplementary material for: How much European prescribing physicians know about invasive fungal infections management?
Source: BMC Infect Dis. 2015 Feb 21;15:80. doi: 10.1186/s12879-015-0809-z (PMC4344747; doi:10.1186/s12879-015-0809-z)
Supplement: Additional file 1: — Consists in the 20- point survey administered to participants. [file 12879_2015_809_MOESM1_ESM.pdf]

EUROPEAN COLLABORATON IN MYCOLOGY (E-COMIC)

SURVEY FOR ANTIFUNGAL PRESCRIBERS

This is 20-questions anonymous survey designed to assess the knowledge on invasive fungal infection and to identify what is the diagnostic approach and antifungal treatment on clinical practice. It is aimed towards attending physicians and residents of the departments with the biggest antifungal prescription volume.

HOSPITAL ____

COUNTRY ____

LOCAL COORDINATOR __________________________________

Data provided in the questionnaire will remain anonymous

Age ☐

Doctor ☐
1. Medical staff
2. Resident
3. Rotating resident

Postgraduate Years ☐

Department ☐
1. Critical care units
2. Medical departments
3. Surgical departments
4. Pediatrics
5. Others

Sex ☐
1. Male
2. Female

1. When *Candida* is isolated in a urine culture, choose the answer that best describes what you would do:

☐

a) Start antifungal treatment in all cases.
b) Start antifungal treatment if the colony count is $>10^4$ cfu/ml.
c) Start antifungal treatment if the colony count is $>10^5$ cfu/ml.
d) Start antifungal treatment if the patient has an indwelling bladder catheter.
e) **Start antifungal treatment only in some cases.**

2. On a patient with mechanical ventilation and a probable Ventilator Associated Pneumonia (VAP), the tracheal aspirate culture shows *Candida* sp.
Which of the following statements best reflect your interpretation?

☐

a) It is obviously colonization and doesn't require antifungal treatment.
b) Requires treatment with fluconazole, since it is a VAP due to *Candida*.
c) Requires treatment with a candin, since it is a VAP due to *Candida*.
d) **Requires antifungal treatment only if the patient has a high *Candida* score.**
e) Requires antifungal treatment if the *Candida* count is $>10^5$ cfu/ml.

3. In which of the following clinical scenarios you would start anti-*Candida* prophylaxis?

a) ICU patient colonized by *Candida*.
b) ICU patient not colonized by *Candida* but having an indwelling bladder catheter, a central venous catheter and recent surgery.
c) Liver transplant recipients.
d) **Acute Myeloid Leukemia patients on induction chemotherapy.**
e) All of the above.

4. In your opinion, the best choice for *Candida* prophylaxis is:

a) **Fluconazole in most of the cases**.
b) Candins.
c) Liposomal Amphotericin B.
d) Voriconazole.
e) None of the above.

5. In a patient with sepsis possibly caused by a femoral catheter infection, you would prescribe:

a) Treatment against Gram positive bacteria.
b) Treatment against Gram positive and Gram negative bacteria.
c) **Treatment against Gram positive and Gram negative bacteria and yeasts.**
d) Treatment against Gram negative bacteria.
e) None of the above. I would remove the catheter and wait to see the culture results before starting therapy.

6. Microbiology informs you that there are yeasts in the Gram stain of a blood culture.

a) **You start antifungal treatment immediately.**
b) You wait to see in how many bottles they grow.
c) You wait to see the final microbiological identification.
d) You remove the catheters and take new blood cultures.
e) You request a cryptococcal serology.

7. In a patient with candidemia, which antifungal would be your first choice before knowing the *Candida* species?

a) I would wait for the full identification of the microorganism before starting any antifungal.
b) Voriconazole.
c) **Fluconazole or a candin.**
d) Liposomal Amphotericin B.
e) Posaconazole.

8. Choose the right answer among the following statements:

a) *Candida glabrata* can become resistant to fluconazole.
b) *Candida krusei* is always resistant to fluconazole.
c) *Candida parapsilosis* is associated to catheter infection.
d) *Candida albicans* is usually susceptible to fluconazole.
e) **All of the above.**

9. During the follow-up of candidemic patients, it is advised to:

a) Get blood cultures after 3-7 days of antifungal treatment.
b) Exclude infective endocarditis by transesophageal echocardiography.
c) Consider sequential treatment switching to an oral azole.
d) Perform an eye fundus examination.
e) **All of the above are true.**

10. In the treatment of candidemia by a fluconazole-susceptible *Candida*, you would usually prescribe:

a) Caspofungin 70 mg on the first day and then 50 mg daily.
b) Fluconazole 200 mg daily.
c) **Fluconazole 400 to 800 mg per day depending on the *Candida* species.**
d) A dosage over 800 mg of fluconazole daily.
e) None of the above.

11. Which is the percentage of fluconazole resistance in *Candida* strains isolated from blood cultures at your hospital in your opinion?

a) **Less than 5%**
b) Between 5 and 10%
c) Between 10 and 20%
d) Between 20 and 30%
e) Over 30%

12. In which of the following scenarios would you choose Amphotericin B as your first choice?

a) In the empirical treatment of candidemia.
b) In a proven invasive aspergillosis.
c) **In unspecified invasive filamentous fungal infection.**
d) In patients intolerant to fluconazole.
e) In infections due to fluconazole resistant *Candida*.

13. Regarding therapy with azoles and candins, which of the following statement is true?

a) **Candins can be used as empirical treatment before knowing the antifungal susceptibility of yeasts.**
b) Voriconazole is used to treat infections due to fluconazole-resistant *Candida* and is preferred to a candin.
c) Voriconazole does not have significant interactions with other drugs so it is preferred to a candin.
d) Candins are superior to fluconazole in the treatment of candidemia due to fluconazole-susceptible species.
e) Posaconazole is the first choice treatment for fluconazole-resistant *Candida*.

14. When *Aspergillus* spp. is recovered from a respiratory sample, you consider that:

a) Antifungal treatment should always be started.
b) Antifungal treatment should always be started in immunosuppressed patients.
c) Treatment should be only provided to patients with compatible radiological findings in the thoracic CT scan.
d) Treatment should be only provided when the fungal invasion is confirmed by biopsy.
e) **Treatment should be only provided to patients who fulfill criteria of proven or probable invasive aspergillosis.**

15. Which of the following statements regarding the Galactomannan test is false:

a) It is highly sensitive for neutropenic patients with invasive aspergillosis (cut-off > 0.5).
b) The test has a low sensitivity in solid organ transplant recipients.
c) Patients receiving some antibiotics could have false positive test results.
d) It helps in the antifungal treatment's follow-up.
e) **It can only be performed in serum samples.**

16. Which of the following are considered radiological findings suggestive of invasive aspergillosis?

a) Presence of dense, well-circumscribed lesions with or without halo sign in a thoracic CT scanner.
b) Presence of a cavity in a thoracic CT scanner.
c) Presence of an air-crescent sign in a thoracic CT scanner.
d) Sinusitis.
e) **All of the above are true.**

17. In a patient with invasive pulmonary aspergillosis, which antifungal treatment would you choose before having the antifungal susceptibility data?

a) **Voriconazole.**
b) Voriconazole + caspofungin.
c) Liposomal Amphotericin B 3 mg/kg/daily.
d) Amphotericin B + voriconazole.
e) Liposomal Amphotericin B 10mg/kg/daily.

18. In your opinion, which are the indications of combined antifungal therapy in invasive aspergillosis?

a) Invasive pulmonary aspergillosis in patients with COPD.
b) Invasive pulmonary aspergillosis in neutropenic patients or transplant recipients.
c) It is not sufficiently supported by clinical evidence.
d) In cases caused by non-*fumigatus Aspergillus* species.
e) **It is only used as rescue therapy, when previous antifungal treatment has failed.**

19. Which is your opinion concerning the measurement of antifungal levels?

a) Up-to-date guidelines do not recommend its systematical determination.
b) They can be useful to identify azoles under-dosed patients.
c) There is no indication to determine serum levels of Liposomal Amphotericin B.
d) It can help to identify azole-related toxicity.
e) **All of the above are true.**

20. In your opinion, which would be the proper length of treatment for solid organ transplant recipients with invasive aspergillosis?

a) 2 weeks.
b) 4-6 weeks.
c) **A minimum of 6-12 weeks.**
d) Three to six months.
e) More than six months.

Thanks for your collaboration.

Would you be interested in participating in an online course on fungal infections?

YES ☐ NO ☐
